# Simple Synthesis of CuInS_2_/ZnS Core/Shell Quantum Dots for White Light-Emitting Diodes

**DOI:** 10.3389/fchem.2020.00669

**Published:** 2020-08-25

**Authors:** Huimin Li, Xiaohong Jiang, Anzhen Wang, Xiaotian Chu, Zuliang Du

**Affiliations:** Key Lab for Special Functional Materials, Ministry of Education, National & Local Joint Engineering Research Center for High-Efficiency Display and Lighting Technology, Collaborative Innovation Center of Nano Functional Materials and Applications, School of Materials Science and Engineering, Henan University, Kaifeng, China

**Keywords:** light-emitting diodes, CuInS_2_/ZnS, core/shell quantum dots, solvothermal synthesis, WLEDs

## Abstract

In this study, the CuInS_2_/ZnS core/shell quantum dots (QDs) were prepared via simple and environmentally friendly solvothermal synthesis and were used as phosphors for white light-emitting diodes (WLEDs). The surface defect of the CuInS_2_ core QDs were passivated by the ZnS shell by forming CuInS_2_/ZnS core/shell QDs. By adjusting the Cu/In ratio and the nucleation temperature, the photoluminescence (PL) peak of the CuInS_2_ QDs was tunable in a range of 651–775 nm. After coating the ZnS layer and modifying oleic acid ligands, the PL quantum yield increased to 85.06%. The CuInS_2_/ZnS QD powder thermal stability results showed that the PL intensity of the QDs remained 91% at 100°C for 10 min. High color rendering index values (CRI, 90) and correlated color temperature of 4360 K for the efficient WLEDs were fabricated using CuInS_2_/ZnS QDs and (Ba,Sr)_2_SiO_4_:Eu^2+^ as color converters in combination with a blue GaN light-emitting diode chip.

## Introduction

Energy shortages and environmental pollution are two major global problems. Lighting requires approximately 19% of all global power consumption. Phosphor-converted white light-emitting diodes (WLEDs) are widely used because they are cost-effective and environmentally friendly. Their commercialization is expanding to large-scale utilization, including display and general lighting. WLEDs have the advantages of energy savings, long lifetimes, compactness, and environmental protection (Du et al., 2016; Kim et al., 2017; Wang et al., 2017; Li et al., 2019; Shen et al., 2019). Yellow Y_3_Al_5_O_12_:Ce^3+^(YAG:Ce) or green light-emitting (Ba,Sr)_2_SiO_4_:Eu^2+^ is often used as a converter phosphor to form WLEDs in combination with InGaN or GaN blue chips (Bachmann et al., 2009; Ji et al., 2016; Dong et al., 2019). However, WLEDs with a low color rendering index (CRI) have some drawbacks, including the use of expensive rare-earth materials and lack of red spectral regions. Therefore, developing novel materials for low-cost, high-efficiency, and high-CRI WLEDs has attracted considerable attention.

Quantum dots (QDs) with color tenability and high luminescence efficiency have significant potential for next-generation indoor lighting and displays (Li et al., 2011; Yu et al., 2018; Du et al., 2019; Song et al., 2019; Moon and Chae, 2020). The II–VI (Cd-based) QDs have been thoroughly studied to be potentially toxic to the environment and humans (Chuang et al., 2014). For the other QDs, such as III–V (InP) (Zhang et al., 2019, 2020), and ternary I–III–VI, such as Cu–In–S (CIS) (Gromova et al., 2017; Chen et al., 2018; Berends et al., 2019; Wegner et al., 2019), Cu–In–Se (Allen and Bawendi, 2008; Houck et al., 2019), quaternary Zn–Cu–In–S (Liu et al., 2015; Dai et al., 2017), and Ag–In–S (Ko et al., 2017) have been studied to replace the traditional Cd-based QDs. The CIS QDs have tunable emission wavelengths, greater stoke shifts, low toxicity, and low cost, which meets the requirements of down-conversion materials. The larger Stokes shift in CIS-based QDs reduces their self-absorption loss, which is beneficial for the performance of light-converting applications. This advantage makes them potential alternatives for color-converting materials. To date, only a few synthetic methods have been used to prepare the CIS-based QDs. On the other hand, the photoluminescence quantum yield (PLQY) of these QDs is not enough for use as phosphor-converted materials. To fully explore the behavior of CIS-based QDs in lighting applications, it is necessary to produce a sufficient amount of QDs and conduct device exploration systematically. The CIS QDs are mainly synthesized by solvothermal method and hot injection. The former methods have advantages of easy preparation and large-scale production. Similar to Cd-based QDs, the surface of the CIS core was often passivated by a ZnS shell for improving the fluorescence of QDs (Li et al., 2011; Kim et al., 2016; Huang et al., 2017). For instance, the Li group increased the PLQY of the CIS QDs up to 10-fold by coating ZnS shell material with a “core/shell” structure; the overgrowth of as-prepared nanocrystals with a few monolayers of ZnS or CdS increases the PLQY exceeding 80% (Li et al., 2011). This ZnS shell cladding is a commonly used method for preparation of core-shell QDs. By the hot injection method, Kim et al. achieved luminescent CIS/ZnS core-shell QDs successfully with PLQY of 65% (Park and Kim, 2011). Yang et al. also obtained high PLQY of 89% CIS/ZnS QDs from a shelling perspective (Kim and Yang, 2016).

By combination with blue-emitting chips, researchers have also tried to do something to fabricate the WLEDs based on CIS-based QDs. For example, Wang et al. fabricated warm WLEDs by adding CIS/ZnS into YAG:Ce silicone with an InGaN-based blue LED chip (Li et al., 2019). Yang et al., using a facile, large-scalable solvothermal method, synthesized CIS QDs with different Cu/In molar ratios and changed the nucleation time via a hot colloidal route (Song and Yang, 2012; Jang et al., 2013). They also obtained a solid-state lighting device with CRI of 70–72 and correlated color temperature (CCT) of 5950–6150 K based on high-PLQY yellow CIS/ZnS QDs of 92% (Song and Yang, 2013). Zhong et al. successfully explored the possibility for light-emitting color-converting materials by mixing green- and red-emissive CIS-based QDs with PLQY of 60–75%, which get a tunable CCT of 4,600–5,600 K with CRI of 95 (Chen et al., 2013). Dual emissive (Mn,Cu) doped Zn–In–S/ZnS QDs were also used as color-converters with PLQY of 75%, and the as-fabricated WLEDs showed bright white light with CRI of 95 and CCT of 5,092 K (Yuan et al., 2015). Besides, Liu's group followed this solvothermal route by varying Cu/In ratios and obtained efficient white light-emitting diodes with high CRI of 90 and CCT of 6,552 K which were fabricated (Chuang et al., 2014). However, in the main, the underdeveloped synthesis for CIS-based QDs hindered its full potential application.

According to the above reports, it has been proved that the white light devices based on CIS/ZnS QDs cannot achieve low CCT with high CRI simultaneously. However, WLEDs with high CRI of 90 and CCT of 4,360 K were fabricated by CIS/ZnS QDs based on blue GaN light-emitting diode (LED) chips in this report. Compared with the most similar literature report with high CCT of 6,552 K (Chuang et al., 2014), this CCT is relatively low at the same CRI of 90. The detailed CIS QDs were synthesized by the solvothermal method with adjusting Cu/In molar ratios. The CIS QDs show tunable emission colors between 651 and 775 nm, which resulted from the different Cu/In ratios for CIS synthesis. Subsequently, the ZnS shell was coated for passivating the core CIS QDs. After the optimization for zinc source and OA ligands with a Cu/In ratio of 1:2, the PLQY of CIS/ZnS QDs can be increased to 85.06%.

## Materials and Methods

### Materials

Copper iodide (CuI, 99.999%), indium acetate (In(OAc)_3_, 99.99%), 1-dodecanethiol (DDT, 98%), 1-octadecene (ODE, 90%), oleic acid (OA, 90%), and zinc acetate (Zn(OAc)_2_, 99.99%) were purchased from Aldrich. All chemicals were used as received.

### Synthesis of CIS/ZnS Nanocrystals

A solvothermal method was employed to synthesize CIS core QDs. In the experiment, a series of Cu/In composition (Cu/In =1:3, 1:2, 1:1, 2:1) were synthesized with fixed amounts of the sum of Cu and In precursors. Herein, the synthetic process of CIS QDs with Cu/In =1:2 is presented. CuI (0.095 g, 0.5 mmol), In(OAc)_3_ (0.292 g, 1 mmol), and 20 mL of DDT were added into a 50 mL teflon-lined autoclave at room temperature. DDT serves as solvent and S source. Then, the mixed solution was heated to 180°C for 6 h. After completion of the nucleation, the teflon-lined autoclave was cooled to room temperature. Then, the red product of CIS core QDs was synthesized. To improve the stability and fluorescence properties, the core/shell structure of CIS need to be coated by ZnS. A shell stock solution was obtained by dissolving Zn(OAc)_2_ (1.100 g, 6 mmol) in 4 mL of DDT, 8 mL of ODE, and 2 mL of OA, which needs to heat to 160°C for 30 min until the solution is transparent. After that, this shell stock solution was added into the CIS core stock solution. Finally, the mixture was further heated to 200°C and this temperature was kept for 14 h, allowing the ZnS shell to grow. By adding chloroform and excessive acetone into the mixture for several times, the as-prepared CIS and CIS/ZnS QDs were purified.

### Fabrication Phosphor-Based White LEDs

Red-emitting CIS/ZnS QDs (at 619 nm) and inorganic green-emitting (Ba,Sr)_2_SiO_4_:Eu^2+^ phosphor particles (G2762, Intermatix Co.) were blended with a UV-curable NOA 61 adhesive. To get a homogeneous mixture, they were put into a vortex for 3,000 rpm/5 min. Subsequently, the mixture of red-emissive CIS/ZnS QDs and the green-emitting (Ba,Sr)_2_SiO_4_:Eu^2+^ phosphor was dropped on a blue LED chip (GaN). Next, the devices were irradiated with 400 W UV light for 20 min.

### Characterization

A Horiba Fluorolog-3 fluorescence spectrometer was used to measure the transient and steady-state photoluminescence (PL) spectra of the samples. A nanoLED diode emitting pulses (405 nm) was employed as an excitation source for the latter measurement. The CIS and CIS/ZnS QDs toluene solution was also diluted for PLQY measurement. The absorption spectra of CIS and CIS/ZnS QDs were investigated by using a UV-vis spectrophotometer (PE Lambda 950). A transmission electron microscope (TEM) operating at an acceleration voltage of 200 kV (JEOL, JEM-2100F) was used to investigate the size and morphologies of the QDs. The high-resolution TEM image was operated by JEOL JEM-ARM200F. Thermal-dependent PL spectra were measured by TAP-02, Orient-KOJI Instrument. X-ray photoelectron spectroscopy (XPS, Thermal 250Xi) was used to measure the compositions of QDs. The electroluminescence (EL) spectra, luminous efficiency, CCT, Commission Internationale de l'Eclairage (CIE) color coordinates, and CRI values of the CIS/ZnS QD-based WLED devices were evaluated under different currents of 10–200 mA in PR-735 at room temperature, respectively.

## Results and Discussion

**Figure 1:**
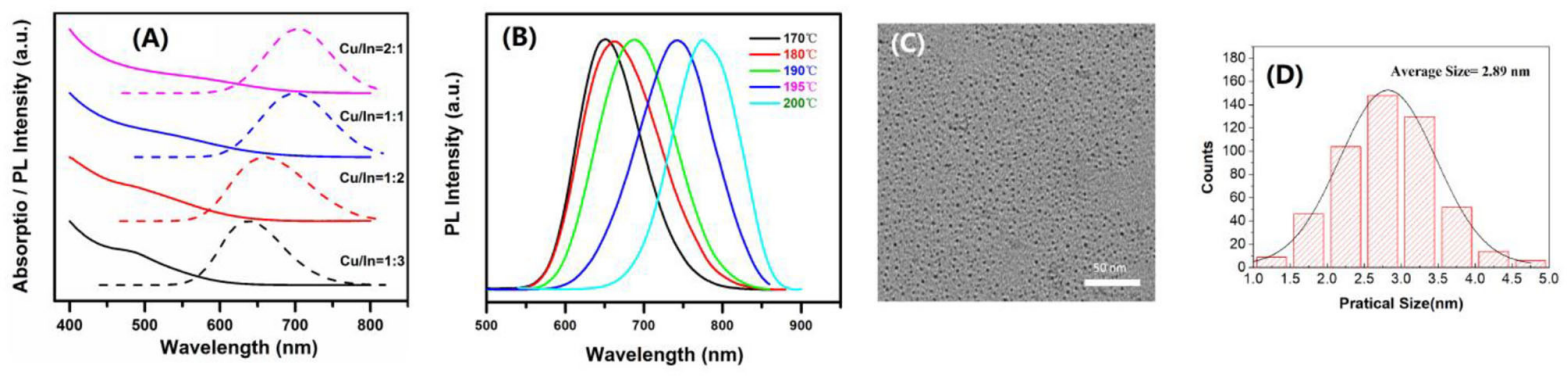
**(A)** Absorption and PL spectra of the CIS core QDs with various Cu/In molar ratios. **(B)** PL spectra with temperature change from 170 to 200°C at the Cu/In molar ratio of 1:2. **(C)** TEM image of CIS core QDs when the Cu/In molar ratio is 1:2 at the nucleation temperature of 180°C. **(D)** The particle size distribution of **(C)**.

The CIS core QDs were prepared by a solvothermal method with different Cu/In ratios. Their absorption spectra are shown in Figure 1A. From this figure, with increasing In ratio, one can see the absorption tends toward a shorter wavelength. There is no obvious exciton absorption peak, which is the common feature of CIS QDs, generally due to broad size distribution or inhomogeneous composition With the increase in In ratio, the emission peak also gradually blue shifts from 704 to 638 nm, resulting in a widened bandgap (Figure 1A). There is also an obvious Stokes shift, for the large difference in absorption of emission-band energies. Usually, the Stokes shift will happen when the excited electron-hole pair (DAP) recombination occurred within the intraband. This recombination is also commonly defined as DAP. With decreasing Cu/In molar ratio from 2:1 to 1:3, the PLQYs of CIS core QDs were investigated to be 4.83, 9.26, 14.22, and 4.05%, respectively. It is obvious that the CIS core QDs have the largest PLQY when the Cu/In molar ratio is 1:2. The nucleation temperature effect on the CIS QDs' PL peak was also studied at the Cu/In molar ratio of 1:2, which is shown in Figure 1B. From this figure, one can see that the PL peak shows a significant red shift from 651 nm to 775 nm with the increase in nucleation temperature. At the nucleation temperature of 180°C with a Cu/In molar ratio of 1:2, a typical low-magnification TEM image is shown in Figure 1C and the related size distribution is shown in Figure 1D. It can be seen that the CIS QDs are in good dispersion with size distribution in 1.4–4.5 nm and the average size is about 2.89 nm.

**Figure 2:**
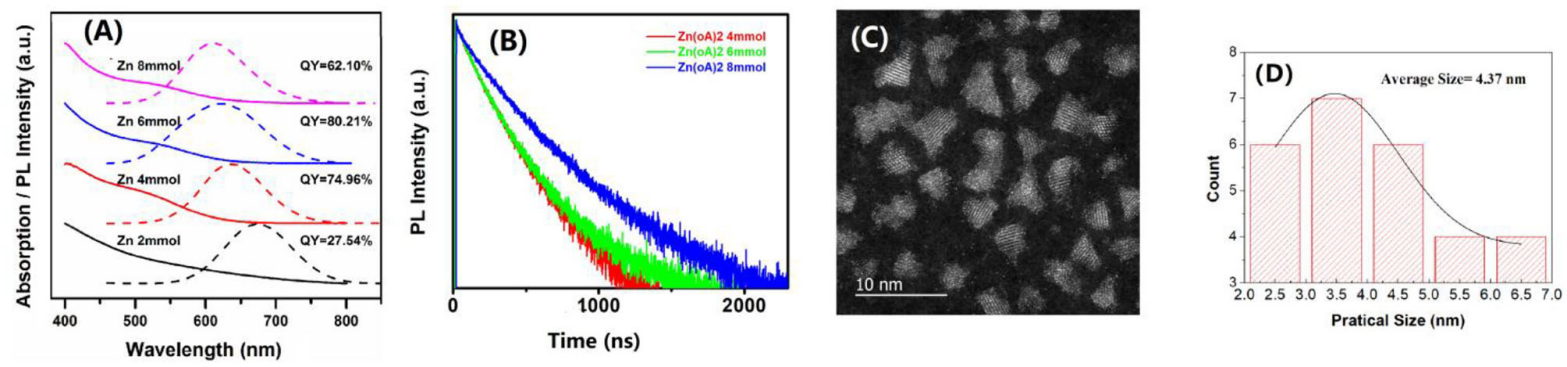
**(A)** Absorption and PL spectra of CIS/ZnS QDs with a Cu/In molar ratio of 1:2 at different uses of zinc acetate of 2, 4, 6, and 8 mmol. **(B)** Time-resolved fluorescence decay traces of CIS/ZnS QDs in **(A)**. **(C)** High-resolution TEM of CIS/ZnS QDs with a Cu/In molar ratio of 1:2, 6 mmol Zn(OAc)_2_, and 4 mL OA. **(D)** The particle size distribution of **(C)**.

Usually, for the case of CIS QDs, there is a high surface-to-volume ratio for its small size. There are too many dangling bonds and more complex lattice defects on the surface of QDs. As a consequence, CIS QDs have very low fluorescence intensity and the maximum quantum yield is approximately 14.22%. To increase the PLQY, the ZnS was coated on the surface of CIS core QDs (Li et al., 2011; Kim et al., 2016; Zhang et al., 2017). The ZnS shell can effectively remove the non-radiation band defects caused by surface defects. Different amounts of zinc acetate were adopted with 2, 4, 6, and 8 mmol, respectively. The optical properties of these CIS/ZnS QDs are shown in Figure 2A. From these PL spectra, it indicated that the PL peak blue shifted from 673 to 609 nm with the increase in zinc acetate. When the zinc acetate was 6 mmol, it got the highest PLQY among these CIS/ZnS QDs, which reached to 80.21% (shown in Figure 2A). It is easy to conclude that the passivation of the bare QDs is not effective when the shell is too thin, resulting in very poor luminescence properties. If the shell is too thick, the lattice strain will be generated by lattice mismatch, accompanied by the formation of defects at the interface of the core and shell. From Figure S1, one can see that the CIS/ZnS QDs increased in size with the increase in zinc acetate, resulting from the gradual thickening of the coated shell. The XRD was further investigated to discuss the CIS/ZnS QD structure with different amounts of zinc acetate, which is shown in Figure S2.

Figure 2B shows a set of room-temperature PL decay profiles of CIS/ZnS QDs with different usages of Zn(OAc)_2_. All decay curves were well fit with a three exponential function with the fitting parameters of relatively fractional contributions (a) and decay time (τ). The average lifetime < τ > was determined according to the following equation:

$$\begin{array}{l} <\tau >={\text{a}}_{1}•\tau_{1}+{\text{a}}_{2}•\tau_{2}+{\text{a}}_{3}\;•\tau_{3} \end{array}$$

The specific fitting parameters of the CIS/ZnS QDs in Figure 2B are shown in Table 1. The average lifetimes of the CIS/ZnS QDs were approximately 377.23, 394.95, and 471.52 ns when the amount of Zn(OAc)_2_ was 4, 6, and 8 mmol, respectively. The increasing lifetimes as the zinc source increased were attributed to the surface passivation of the ZnS shell. In addition to coating the wide-bandgap inorganic ZnS material to passivate the CIS/ZnS QD surface defects, OA was also used to modify the QDs. OA was used as the ligand and eliminated the surface trap-states of the QDs. The correlated TEM images of the CIS/ZnS QDs (Figure S3) show that amounts of OA have little effect on the QD sizes. The XRD peaks of CIS/ZnS QDs with different amounts of OA are similar to Figure S2 as illustrated in Figure S4. The appearances of the stretching vibrations of the carbonyl moiety in the CIS/ZnS QDs at 1559 and 1471 cm^−1^ obtained using Fourier transform infrared spectroscopy (FTIR, Figure S5) proved that the OA was successfully coated on the CIS/ZnS QDs. The PLQY reached 85.06% when 4 mL of OA was used. Compared to the CIS core QDs, the high-resolution TEM image of the CIS/ZnS QDs (Figure 2C) shows that the size distribution was not uniform. The related size distribution of CIS/ZnS QDs (Figure 2D) is wide from 2.0 to 7.0 nm and the average size is about 4.37 nm. On the other hand, although CuInS_2_ and ZnS have different crystal structures, their lattice mismatch is small, so that the ZnS shell is able to grow well on the surface of CuInS_2_ crystals (Li et al., 2019). This small-lattice mismatch probably makes the core-shell structure difficult to observe. So, compared with the core of the CIS QDs, the shape of CIS/ZnS QDs is changed from spherical to irregular, and the average size also increased, which can prove indirectly that the ZnS shell can grow on surface of CuInS_2_ crystals.

**Table 1:** Three exponential fit parameters from Figure 2B.

	**8 mmol Zn(OAc)_**2**_**	**6 mmol Zn(OAc)_**2**_**	**4 mmol Zn(OAc)_**2**_**
a_1_	66.01%	51.57%	74.44%
t_1_ (ns)	295.04	559.18	439.00
a_2_	25.18%	47.59%	25.00%
t_2_ (ns)	1099.09	223.74	200.70
a_3_	8.81%	0.84%	0.56%
t_3_ (ns)	0.25	13.91	50.72
< τ > (ns)	471.53	394.96	377.11

**Figure 3:**
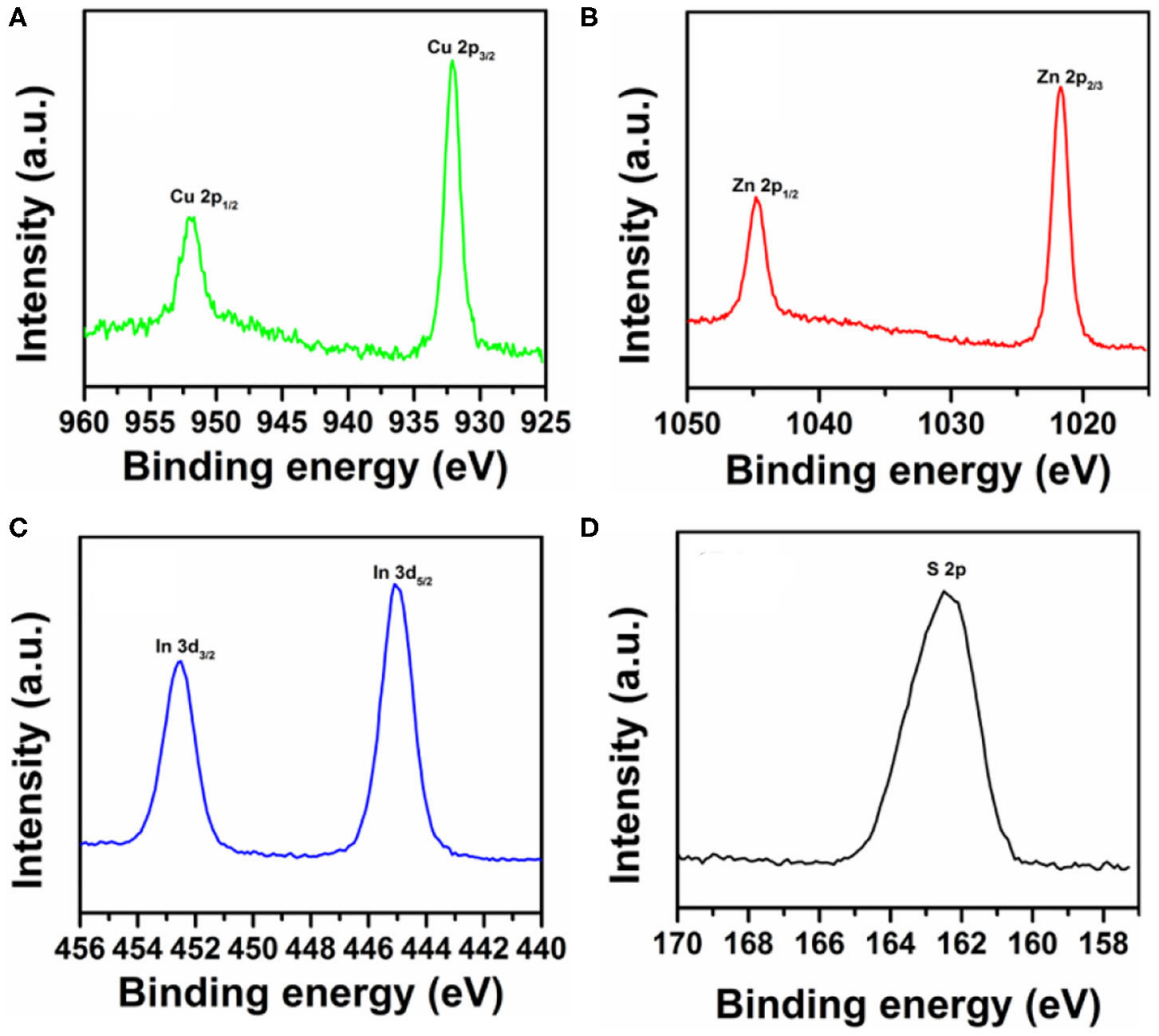
High-resolution XPS spectra of **(A)** Cu 2p, **(B)** Zn 2p, **(C)** In 3d, and **(D)** S 2p.

The chemical compositions of the CIS/ZnS QDs in Figure 2C were further determined via XPS characterization as shown in Figure 3. The high-resolution XPS peaks of In 3d, Zn 2p, Cu 2p, and S 2p are demonstrated (Liu et al., 2015). Figure 3A shows the binding energies of the Cu 2p_3/2_ and Cu 2p_1/2_ peaks at 932.1 and 951.9 eV, respectively. The Zn 2p peaks (1027.7 eV of Zn 2p_3/2_ and 1044.8 eV of Zn 2p_1/2_) occurred due to the presence of the ZnS shell layer as shown in Figure 3B. As demonstrated in Figure 3C, the binding energies at 445.1 and 452.3 eV were attributed to In 3d_5/2_ and In 3d_3/2_, respectively. Figure 3D shows the S 2p XPS spectra and an S 2p peak at a binding energy of 162.4 eV. The elemental composition of the QDs was explained qualitatively by this characterization.

**Figure 4:**
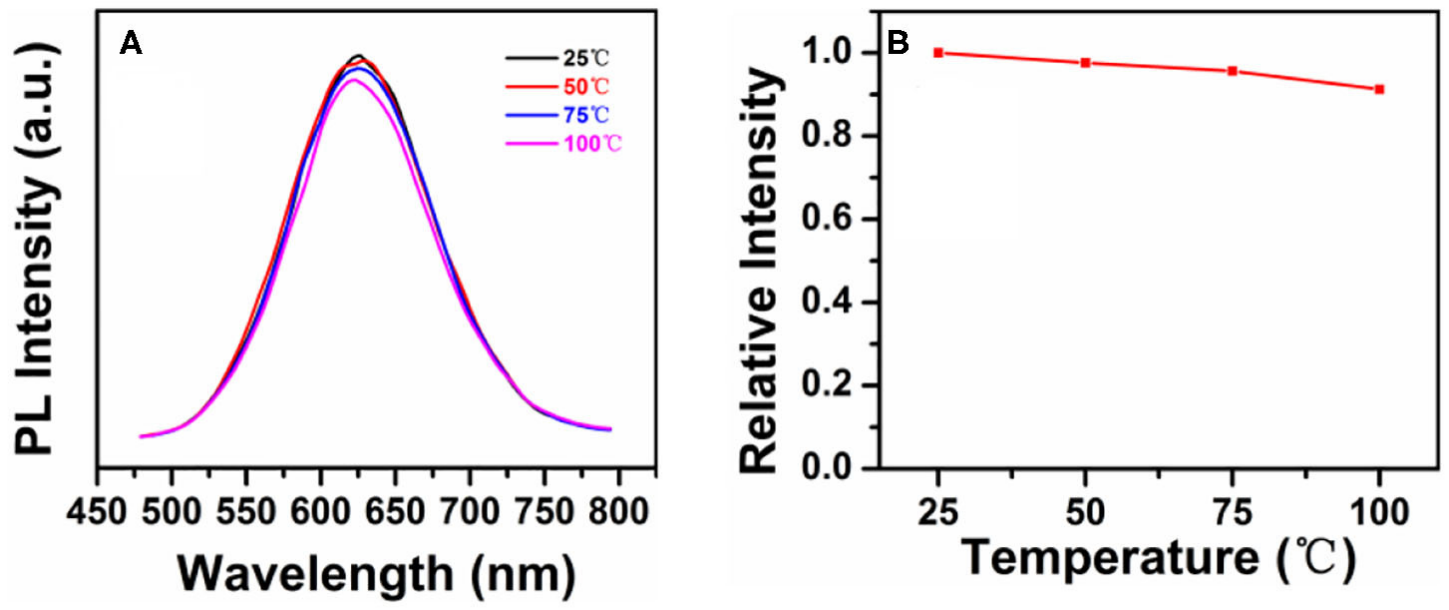
**(A)** PL spectra of the CIS/ZnS QDs at different temperatures from 25 to 100°C under excitation at 450 nm. **(B)** Relative intensity of the QDs in **(A)**.

The luminous efficiency of quantum dots usually decreases gradually in WLEDs at high temperatures. However, in photoelectric devices with QDs as the down-conversion materials, the working temperature is usually higher than room temperature due to P–N junction heating. Therefore, the thermal stability of QDs is essential (Lee et al., 2018; Li et al., 2018). Figure 4 shows the PL spectra of CIS/ZnS QDs in Figure 2C (with OA ligand of 4 mL) at different temperatures. Similar to the wavelength of the blue GaN chips used in the following experiment, an excitation wavelength of 450 nm was used. Figure 4A demonstrates that the PL intensity decreased slightly as the temperature increased from 25 to 100°C. The PL intensity remained approximately 91% at 100°C as shown in Figure 4B, which indicates that the synthesized CIS/ZnS QDs had good thermal stability.

**Figure 5:**
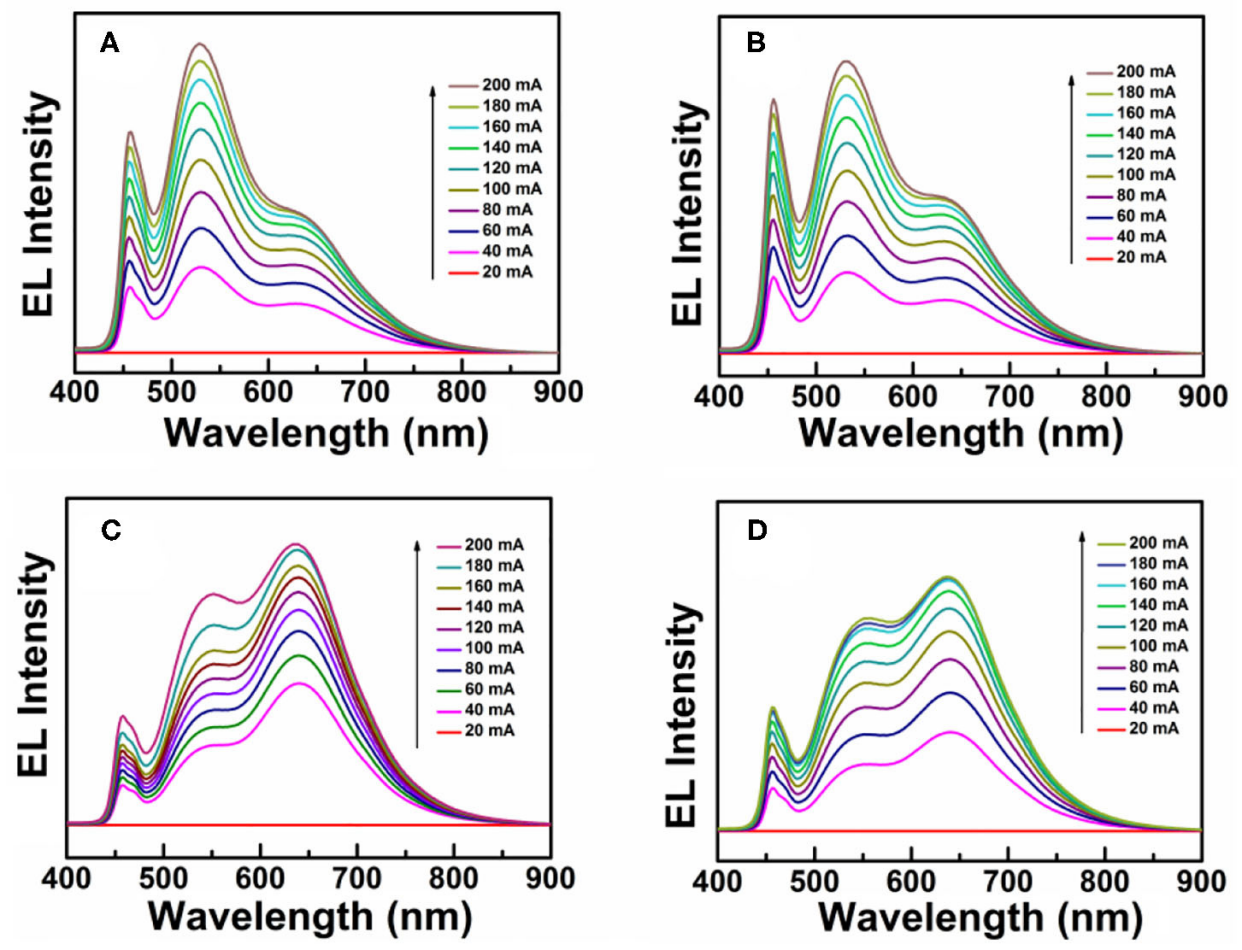
EL spectra with different ratios of CIS/ZnS QDs to green (Ba,Sr)_2_SiO_4_:Eu^2+^ phosphor. **(A)** 1:7, **(B)** 1:6, **(C)** 1:5, and **(D)** 1:4.

Due to intrinsically large Stokes shifts, CIS/ZnS QDs can be used as promising solid-state down-conversion materials. In this study, WLEDs were fabricated with red CIS/ZnS QDs and blue GaN LEDs. CIS/ZnS QDs (red phosphors: R) and (Ba,Sr)_2_SiO_4_:Eu^2+^ phosphors (green phosphors: G) were mixed uniformly with transparent UV resin. Due to the fact that the B source (GaN with 1 W) is fixed, the proportion of R and G should be adjusted through their relative proportion and total amount. Firstly, the relative proportion of R and G was adjusted to fabricate WLEDs. For a typical WLED, 0.5 g of UV resin was blended with 0.09 g of a mixture of CIS/ZnS QDs and(Ba,Sr)_2_SiO_4_:Eu^2+^ phosphors. Four devices were designed with different ratios of CIS/ZnS QDs to green phosphor at a fixed amount of UV resin. The performance of the devices at different working currents is illustrated in Table 2. The CCT decreased as the CIS/ZnS QDs ratio in the mixture of CIS/ZnS QDs and (Ba,Sr)_2_SiO_4_:Eu^2+^ phosphors increased. The CRI of device D reached 89, and the CCT was approximately 3,450 K when the ratio of CIS/ZnS QDs to green phosphors was 1:4. The related EL spectra of the devices at different working currents are illustrated in Figure 5. The 466, 539, and 640 nm bands were caused by the blue GaN chips (Ba,Sr)_2_SiO_4_:Eu^2+^ phosphor, and CIS/ZnS QDs, respectively. The relative peak intensity of the green light region decreased gradually as the proportion of green phosphor progressively decreased. Compared to the EL peak of 466 nm, the EL peak of the CIS/ZnS QDs and (Ba,Sr)_2_SiO_4_:Eu^2+^ increased as the CIS/ZnS QDs ratio increased. Especially for device D, the EL peaks of the 539 and 640 nm bands were much stronger than at 466 nm. It is obvious that when the ratio of R to G is 1:4, the device's CRI is 89 and the B proportion is much lower than that of R and G.

**Table 2:** Four WLEDs with different ratios of CIS/ZnS QDs to green (Ba,Sr)_2_SiO_4_:Eu^2+^ phosphor and their performance.

**Device**	**UV resin (g)**	**UV:(R+G)**	**R:G**	**CIE (x, y)**	**CCT (K)**	**CRI**
A	0.5	0.5:0.09	1:7	(0.325, 0.436)	5,708	73
B	0.5	0.5:0.09	1:6	(0.331, 0.417)	5,564	79
C	0.5	0.5:0.09	1:5	(0.417, 0.446)	3,653	84
D	0.5	0.5:0.09	1:4	(0.423, 0.432)	3,450	89

**Figure 6:**
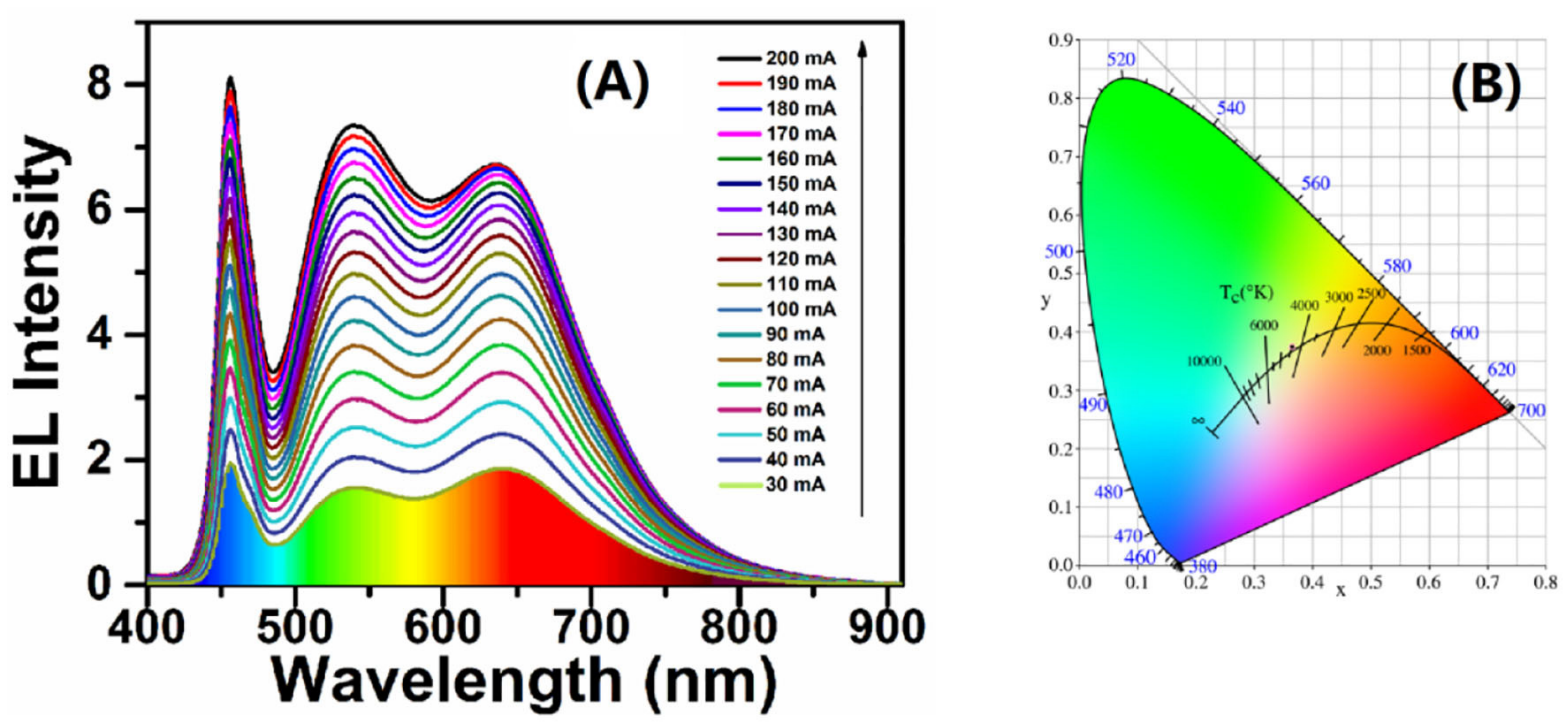
**(A)** Forward current-dependent EL spectral evolution. **(B)** CIE white light color coordinates.

Based on the WLEDs device with high CRI of 89, we tried to reduce the total amount of phosphors under the ratio of CIS/ZnS QDs to green phosphors. Three devices were designed with different ratios of UV resin to total phosphor at a fixed R:G of 1:4. The performance of the devices at different working currents is shown in Table 3. Comparing the CCT and CRI values of the three kinds of WLEDs, device E had the best performance, with 4360 K and 90, respectively. Although device F had a high CRI value of 91, its CCT value was approximately 5337 K, which was much higher than that of device E. Figure 6A shows the EL spectra of device E in Table 3 at different working currents. Similarly, the 466-nm, 539-nm, and 640-nm bands were also attributed to the blue GaN chips, (Ba,Sr)_2_SiO_4_:Eu^2+^ phosphor, and CIS/ZnS QDs, respectively. The EL intensity increased as the working current increased, which also indicated that the WLED was stable. Figure 6B shows that the CIE color coordinate of the WLED with a CCT of 4360 K is at (0.365, 0.375). Optical images of the WLED are shown in Figure S6 under zero bias and a forward current of 20 mA, demonstrating that white light was obtained. This shows that warm white light can be obtained by combining CIS/ZnS QDs and (Ba,Sr)_2_SiO_4_:Eu^2+^ phosphors with blue GaN chips. After optimization using UV curable resin, red CIS/ZnS QDs, and commercial (Ba,Sr)_2_SiO_4_:Eu^2+^ phosphors, the CCT of the WLEDs reached 4,360 K with a CRI of 90 at a CIE of (0.365, 0.375).

**Table 3:** Three WLEDs with different ratios of CIS/ZnS QDs to green (Ba,Sr)_2_SiO_4_:Eu^2+^ phosphor and their performance.

**Device**	**UV resin (g)**	**UV:(R+G)**	**R:G**	**CIE (x, y)**	**CCT (K)**	**CRI**
E	0.5	0.5:0.08	1:4	(0.365, 0.375)	4,360	90
F	0.5	0.5:0.07	1:4	(0.338, 0.384)	5,337	91
G	0.5	0.5:0.06	1:4	(0.291, 0.368)	7,206	80

## Conclusion

Different types of CIS/ZnS QDs were successfully prepared using varying zinc sources and ligands via solvothermal synthesis. The surface defects of the CIS core QDs were passivated by a ZnS shell, and their PLQY reached 85.06% with an optimized zinc source and OA ligands with a Cu/In ratio of 1:2. As the ZnS shell thickness increased, the CIS/ZnS QDs had a blue-shifted PL peak. The CIS/ZnS QDs films' thermal stability showed that the PL intensity of the QDs remained 91% at 100°C for 10 min. Efficient WLEDs with a CCT of 4,360 K and a maximum CRI of 90 were fabricated by combining CIS/ZnS QDs with (Ba,Sr)_2_SiO_4_:Eu^2+^ phosphor as color converters with blue GaN-based LED chips.

## Data Availability Statement

All datasets generated for this study are included in the article/Supplementary Material.

## Author Contributions

XJ and ZD planned and programmed all experiments. HL conducted most of the experiments in the lab. XJ wrote the first draft and final manuscript including the discussion. AW performed the WLED test and XC helped with the final manuscript. All authors contributed to the article and approved the submitted version.

## Conflict of Interest

The authors declare that the research was conducted in the absence of any commercial or financial relationships that could be construed as a potential conflict of interest.
