# Supplementary material for: Simple Synthesis of CuInS2/ZnS Core/Shell Quantum Dots for White Light-Emitting Diodes
Source: Front Chem. 2020 Aug 25;8:669. doi: 10.3389/fchem.2020.00669 (PMC7477729; doi:10.3389/fchem.2020.00669)
Supplement: Supplementary file 1 [file Table_1.docx]

Supplementary Material

**CuInS_2_/ZnS Core/Shell Quantum Dots Characterization.** The crystal structure of the QDs was analyzed by X-ray powder diffraction (XRD) spectroscopy using D8-ADVANCE. The size and morphologies of the QDs were investigated by JEM-2100F transmission electron microscopy (TEM) operating at an acceleration voltage of 200 kV, the TEM samples were obtained by depositing a drop of QDs dispersion in chloroform onto carbon coated copper grid network. Fourier transformed infrared (FTIR) spectroscopy (Frontier IR/FIR STA 8000) were used to investigate the dried pure CuInS_2_/ZnS QDs powder at different use of OA.


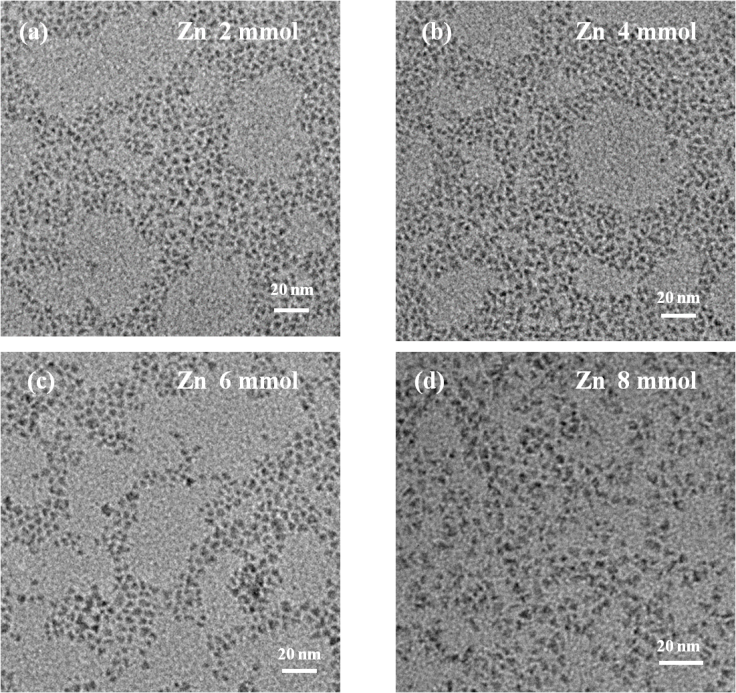


**Figure S1.** TEM image of CIS/ZnS QDs under the different use of zinc source. (a) 2 mmol; (b) 4 mmol; (c) 6 mmol; (d) 8 mmol.





**Figure S2.** XRD patterns of CIS/ZnS QDs at different use of zinc source of 2 mmol, 4 mmol, 6 mmol and 8 mmol.

Three main peaks of (111), (220) and (311) planes were positioned aside from the reflection peaks of CIS/ZnS phase and thus indicating that the ZnS overlayer was deposited appropriately on the surface of CIS core QDs (ZnS, JPDS 05-0566; CIS, JPDS 85-1575).


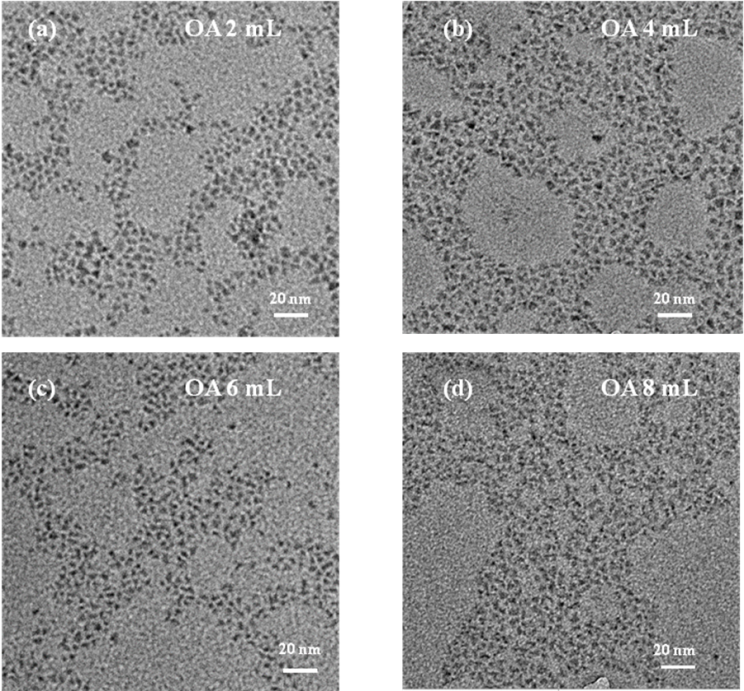


**Figure S3.** TEM image of CIS/ZnS QDs under different use of OA. (a) 2 mL; (b) 4 mL; (c) 6 mL; (d) 8 mL.

c


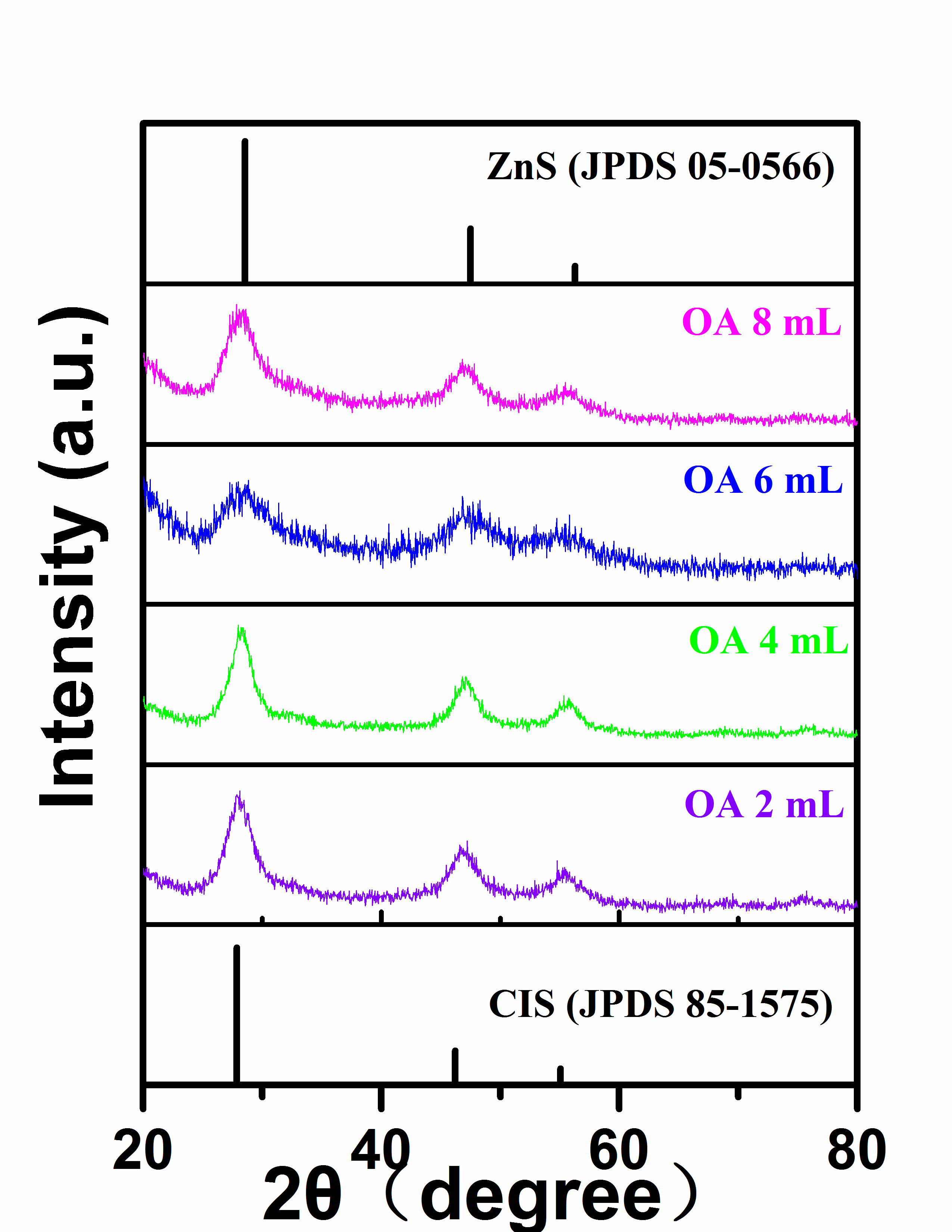


**Figure S4.** XRD patterns of CIS/ZnS QDs at different use of OA of 2 mL, 4 mL, 6mL and 8 mL.


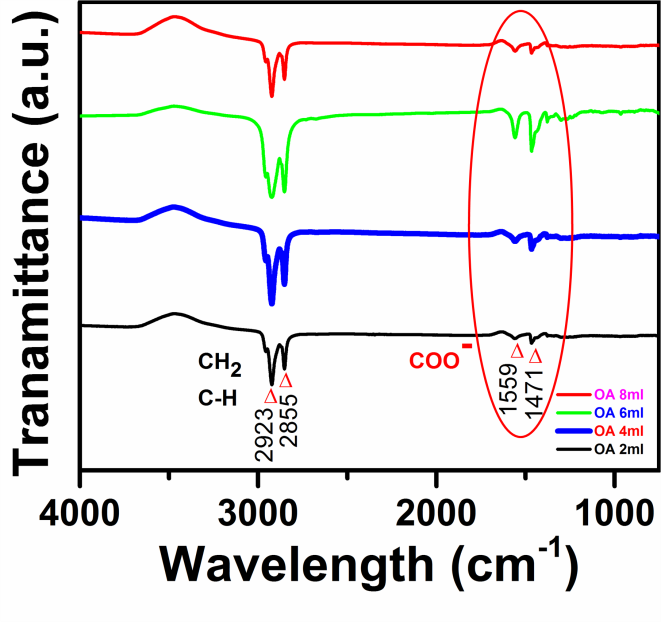


**Figure S5.** FTIR spectra of CuInS_2_/ZnS QDs at different use of OA.


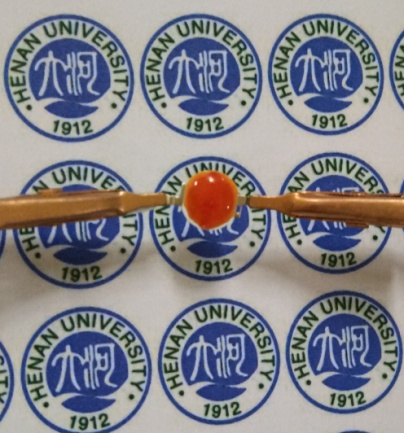

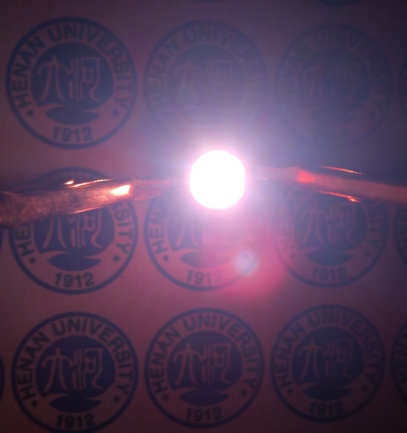


**Figure S6.** Digital camera images of fabricated device under zero bias (left) and forward bias 20 mA (right).
